# Formation of γH2AX and pATM Foci in Human Mesenchymal Stem Cells Exposed to Low Dose-Rate Gamma-Radiation

**DOI:** 10.3390/ijms20112645

**Published:** 2019-05-29

**Authors:** Stepan Ulyanenko, Margarita Pustovalova, Sergey Koryakin, Evgenii Beketov, Anatolii Lychagin, Liliya Ulyanenko, Andrey Kaprin, Anna Grekhova, Alexandra M. Ozerova, Ivan V. Ozerov, Natalia Vorobyeva, Peter Shegay, Sergey Ivanov, Sergey Leonov, Dmitry Klokov, Andreyan N. Osipov

**Affiliations:** 1 A. Tsyb Medical Radiological Research Centre—Branch of the National Medical Research Radiological Centre of the Ministry of Health of the Russian Federation, Koroleva 4, Obninsk 249030, Russia; ustev@mail.ru (S.U.); korsernic@mail.ru (S.K.); beketov.ee@yandex.ru (E.B.); lychagin1@yandex.ru (A.L.); oncourolog@gmail.com (S.I.); 2 State Research Center—Burnasyan Federal Medical Biophysical Center of Federal Medical Biological Agency, Moscow 123098, Russia; pu.margo@mail.ru (M.P.); annagrekhova1@gmail.com (A.G.); varnivey@gmail.com (I.V.O.); nuv.rad@mail.ru (N.V.); 3 Semenov Institute of Chemical Physics, Russian Academy of Sciences, Moscow 119991, Russia; 4 Moscow Institute of Physics and Technology, Dolgoprudny 141700, Russia; leonov.sv@phystech.edu; 5 National Medical Research Radiological Centre of the Ministry of Health of the Russian Federation, Moscow 125284, Russia; kaprin@mail.ru; 6 Emanuel Institute for Biochemical Physics, Russian Academy of Sciences, Moscow 119991, Russia; 7 Faculty of Biology, Lomonosov Moscow State University, GSP-1, Leninskie Gory, Moscow 119991, Russia; ozerova.am@yandex.com; 8 Center for Innovative Radiological and Regenerative Technologies of the Ministry of Health of the Russian Federation, Koroleva 4, Obninsk 249030, Russia; dr.shegai@mail.ru; 9 Institute of Cell Biophysics, Russian Academy of Sciences, Institutskaya St., 3, Pushchino 142290, Russia; 10 Department of Biochemistry, Microbiology and Immunology, University of Ottawa, Ottawa, ON K1N 6N5, Canada; dmitry.klokov@cnl.ca

**Keywords:** DNA double-strand breaks, γH2AX, pATM, γ-radiation, low dose-rate, human mesenchymal stem cells

## Abstract

DNA double-strand breaks (DSB) are among the most harmful DNA lesions induced by ionizing radiation (IR). Although the induction and repair of radiation-induced DSB is well studied for acute irradiation, responses to DSB produced by chronic IR exposures are poorly understood, especially in human stem cells. The aim of this study was to examine the formation of DSB markers (γH2AX and phosphorylated kinase ATM, pATM, foci) in human mesenchymal stem cells (MSCs) exposed to chronic gamma-radiation (0.1 mGy/min) in comparison with acute irradiation (30 mGy/min) at cumulative doses of 30, 100, 160, 240 and 300 mGy. A linear dose-dependent increase in the number of both γH2AX and pATM foci, as well as co-localized γH2AX/pATM foci (“true” DSB), were observed after an acute radiation exposure. In contrast, the response of MSCs to a chronic low dose-rate IR exposure deviated from linearity towards a threshold model, for γH2AX, pATM foci and γH2AX/pATM foci, with an indication of a “plateau”. The state of equilibrium between newly formed DSB at a low rate during the protracted exposure time and the elimination of a fraction of DSB is proposed as a mechanistic explanation of the non-linear DSB responses following a low dose-rate irradiation. This notion is supported by the observation of the elimination of a substantial fraction of DSB 6 h after the cessation of the exposures. Our results demonstrate non-linear dose responses for γH2AX and pATM foci in human MSCs exposed to low dose-rate IR and showed the existence of a threshold, which may have implications for radiation protection in humans.

## 1. Introduction

For the estimation of long-term stochastic radiation health effects, such as cancer, and for radiation protection purposes, a linear-non-threshold (LNT) model extrapolating the effects of high to low doses (≤100 mGy) is applied [1]. To account for the sparring effect seen after low dose-rate exposures (≤0.1 mGy/h), a Dose and Dose-Rate Effectiveness Factor (DDREF) of 2 is used [2]. The radiobiological effects and underlying mechanisms triggered by acute ionizing radiation (IR) exposures have been studied comprehensively. However, the most common IR exposure mode encountered by humans both in the environment and occupationally is protracted or chronic low dose-rate exposure. Current knowledge on the biological effects of such IR exposures is limited and contradictory. The effects of acute vs. chronic IR exposure on the fundamental biological processes, such as DNA repair, cell death, epigenetic changes, carcinogenesis, and aging are the main source of the controversies. DNA double-strand breaks (DSB) are the most deleterious lesions elicited by IR in the DNA of eukaryotic cells. The incorrect repair of DSB often leads to cell death or changes in a genome that can increase the risk of cancer [3,4,5]. Non-homologous end joining (NHEJ) and homologous recombination (HR) are the main pathways of DSB repair. The efficiency and accuracy of DSB repair in somatic cells after acute and chronic IR exposures still remains contradictory [6,7]. About 70–80% of all DSB caused by sparsely ionizing radiation are repaired by the rapid but error-prone NHEJ pathway [8]. Although dose-rate influences the radiobiological effects and cancer risks [9], whether the efficiency and accuracy of DSB repair depends on dose-rate is still unclear.

The studies of the effects of low-dose and low dose-rate IR exposures on DSB repair in multipotent mesenchymal stem/stromal cells (MSCs) are of particular importance because the high proliferative potential of MSCs creates a risk of a transmission of accumulated DNA damage and mutations to both the self-renewed stem cell pool and the differentiated progeny of exposed cells [10,11]. Under normal conditions, MSCs exert suppressive effects on cancer cells [12]. However, there is little doubt that MSCs can also substantially contribute to tumor development and progression [13]. The generation of cancer stem cells (CSCs), epithelial-to-mesenchymal transition (EMT), angiogenesis, drug resistance, and metastasis can all be affected and mediated by MSCs due to them being a part of the tumor microenvironment and due to their ability to migrate to the tumor site in many cancers, including osteosarcoma [14]. Histone modifications, which are key processes in DNA damage responses, are also involved in the generation of CSCs that are known to contribute to colonization and metastases formation [15]. Interestingly, this can be accompanied or mediated by the mesenchymal-to-epithelial transition (MET) of the CSCs, which has also been observed during the embryonic development [16]. Mounting evidence suggests a key role of MSCs in MET via the secretion of the hepatocyte growth factor, which has been associated with an aggressive phenotype and poor clinical prognosis in several types of solid tumors [17,18]. Last, since MSCs are being considered the most promising stem cell type for future regenerative medicine therapies [19], the revealed potential of altered MSCs to contribute to malignancies is of great concern and a limiting factor for otherwise efficient regenerative therapies [20,21]. The key question in this regard is therefore whether low-dose rate radiation exposures are capable of producing changes in the MSCs that can cause the transformation of stem cells into CSCs [22]. The initial events triggering such a transformation are traditionally associated with DSB due to their links with chromosomal instabilities or gene mutations [5].

In higher eukaryotic cells, DSBs rapidly initiate the phosphorylation of the histone H2A variant, H2AX, at Serine 139 to generate γH2AX [23]. The ataxia telangiectasia mutated (ATM), ataxia telangiectasia and Rad3-related (ATR) and DNA-dependent protein kinase catalytic subunit (DNA-PKcs), members of the PIKK (phosphatidylinositol 3-kinase-like kinase) family, phosphorylate H2AX within a minute after irradiation [24,25]. ATM, autophosphorylated immediately upon DSB formation (pATM), is the main kinase phosphorylating H2AX [26]. As a result of these phosphorylation processes, thousands of γH2AX, flanking to the sites of DSB, can be visualized by immunofluorescent methods as bright spots called “foci”. The enumeration of γH2AX and phosphoATM foci after DNA-damaging insults has proven to be a sensitive method, not only for the evaluation of the DSB formation, but also for the assessment of DNA repair kinetics [27,28,29].

The objective of this study was to assess the pattern of change in the number of γH2AX and pATM foci in human MSCs during a continuous (up to 50 h) low dose-rate (0.1 mGy/min) γ-radiation exposure in comparison to an acute irradiation at equivalent doses. We also assessed normalized foci numbers per dose unit for the two exposure modes and carried out a fitting of the corresponding dose-response data to compare the linear vs. threshold dose-response models.

## 2. Results

### 2.1. Low γH2AX Foci Yield in MSCs During Chronic Irradiation

**Figure 1:**
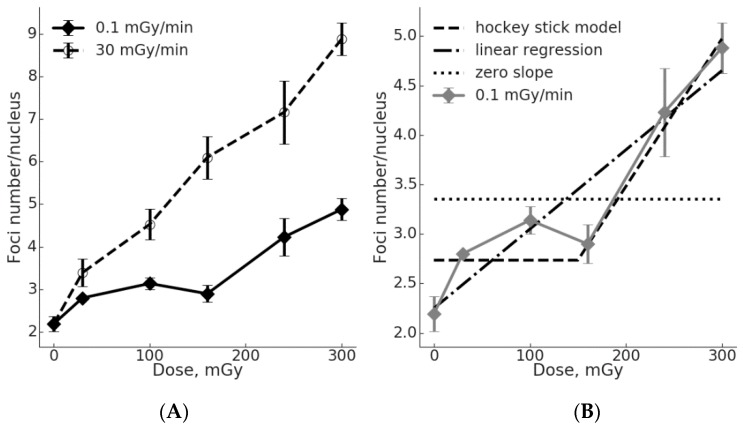
Dose-effect curves for the γH2AX foci formation in γ-irradiated MSCs. (**A**) Data generated using either acute or low dose-rate exposure are shown. Mean and SEM of at least three independent experiments are shown on the *y*-axis. (**B**) Fitting the γH2AX foci data for chronic exposure using a “hockey stick” model (threshold at 150 mGy), a linear regression with a positive slope or a liner regression with a nil slope. See text for details.

The number of γH2AX foci in MSC nuclei was evaluated following chronic (up to 50 h, 0.1 mGy/min) and acute (up to 10 min, 30 mGy/min) γ-radiation exposures at total doses of 30, 100, 160, 240 and 300 mGy (Figure 1). The regression analysis demonstrated a linear dose-response curve y = 2.478 + 0.021x (R^2^ = 0.988; where “y” is number of γH2AX foci, and “x” is the radiation dose in mGy). The data for the chronic radiation exposure can be fitted by a linear regression y = 2.249 + 0.008x (R^2^ = 0.888; where “y” is number of γH2AX foci, and “x” is the radiation dose in mGy). The differences in the number of γH2AX foci between the irradiated and control cells were significant (*p* < 0.05) for all doses of both dose rates, with the exception of 160 mGy at a dose-rate of 0.1 mGy/min.

In the linear regression equation used for the data fitting (y = a + bx), the coefficient “b” reflects a slope of the dose-response curve or the effect per dose unit. It is clearly seen from Figure 1 that the effect per dose unit for the induction of γH2AX foci is higher for acute vs. chronic irradiation (2.6-fold higher). It is reasonable to assume that the γH2AX foci method used captured all or nearly all DSB produced by the acute exposure. In other words, the influence of DSB repair on the scores was minimal. In contrast, lowering the dose rate and protracting the exposure would allow for much more time for DSB repair to complete the repair of slowly occurring DSB, so that by the time of the completion of the exposure (accumulation of the total dose) and fixation of cells, a reduced number of γH2AX foci is observed (Figure 1). Notably, the dose-response curve for the chronic irradiation had a “plateau” between 30 and 160 mGy. The linear model with a zero slope for this dose range cannot be rejected (*p* = 0.28), supporting the notion of the “plateau”. Above 160 mGy, significant increases in the observed number of γH2AX foci affected the goodness of a linear fit, so that the linear hypothesis with a zero slope can no longer be accepted (*p* = 0.007). On the other hand, the linear hypothesis for all six data points still cannot be rejected (*p* = 0.67), similar to the hockey stick hypothesis with a threshold dose of 150 mGy (*p* = 0.72) (Figure 1B).

### 2.2. Comparison of the γH2AX Foci Yields per Radiation Dose Unit for Acute vs. Chronic Exposure

An acute IR exposure led to a pronounced relative increase (I_REL_) in the number of γH2AX foci (irradiation vs. control group) which depended on the dose (Table 1). For a chronic IR exposure at doses 30, 100 and 160 mGy, the value of I_REL_ varied between 1.28 and 1.43. The highest I_REL_ for chronic irradiation was 2.23 at 300 mGy. Of note, the acute irradiation resulted in 1.2–2.1-fold higher I_REL_ values compared to the chronic exposure. The maximal difference in the I_REL_ values (more than 2-fold) between the two types of exposure was observed at 160 mGy.

The absolute yields of DSB per unit of radiation dose (K, %), defined as the increase in the number of γH2AX foci normalized per dose unit, also indicate the significant difference between the acute and chronic exposures (Table 2). The K coefficient for the acute irradiation was higher than that for the chronic exposure for all doses. The maximum difference (5.5-fold) between the two dose-rates was observed at 160 mGy, whereas the minimum difference (2-fold) was found for 30 mGy (Table 2).

### 2.3. The Threshold for ATM Activation upon Chronic Irradiation

**Figure 2:**
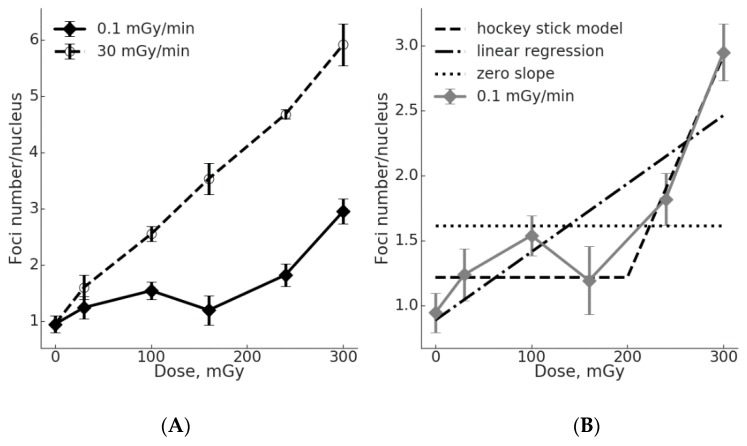
Dose-effect curves for pATM foci formation in γ-irradiated MSCs. (**A**) Data generated using either acute or low dose-rate exposure are shown. The mean and SEM of at least three independent experiments are shown on the *y*-axis. (**B**) Fitting the pATM foci data for chronic exposure using a “hockey stick” model (threshold at 150 mGy), a linear regression with a positive slope or a liner regression with a nil slope. See text for details.

To assess whether ATM may be involved in the γH2AX foci formation, we performed a quantitative analysis of active (phosphorylated) ATM kinase (pATM) foci in MSCs exposed to acute vs. chronic low dose-rate irradiation (Figure 2).

A regression analysis demonstrated a linear fit of the dose-response curve for pATM foci upon acute exposure: y = 0.993 + 0.016x (R^2^ = 0.997; where “y” is number of pATM foci, and “x” is the dose in mGy) (Figure 2A). For the chronic irradiation, the number of pATM foci did not significantly differ from the control within the dose range of 30–160 mGy. Therefore, the linear regression with a nil slope cannot be rejected (*p* = 0.51). At 240 and 300 mGy, the number of pATM foci increased and the linear hypothesis with a nil slope for all 6 experimental data points should be rejected (*p* = 0.003). Additionally, we tested the statistical significance of a “hockey stick” hypothesis with a threshold dose set at 200 mGy and found that it could not be rejected (*p* = 0.95). However, the linear fit for all data points could not be rejected as well (*p* = 0.45) (Figure 2B).

The yields of DSB per unit of radiation dose (K, %) based on the pATM foci formation, were calculated (Table 3). A pattern similar to the one revealed for γH2AX foci was seen again. In particular, small increases were seen for the chronic exposure and significantly higher increases for the acute exposure, up to 10.4-fold and 4.3-fold higher at 160 mGy and 240 mGy, respectively. Therefore, the acute exposure produces a greater number of pATM foci per unit dose in MSCs compared to the chronic exposure.

### 2.4. Co-localization of γH2AX and pATM Foci

**Figure 3:**
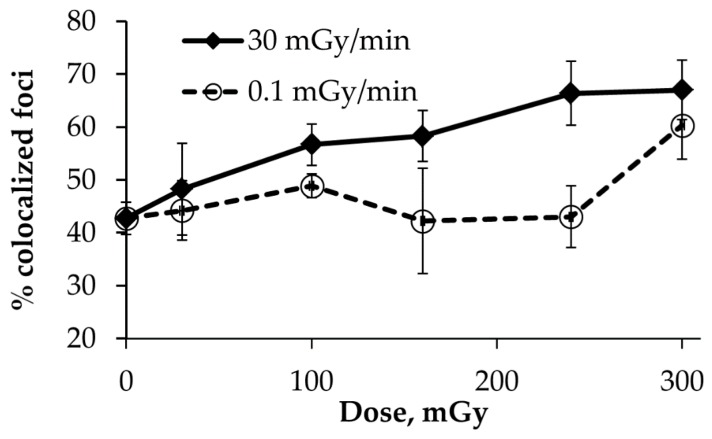
Percentage of γH2AX foci co-localized with pATM foci in MSCs exposed either acutely or chronically to γ-radiation. The mean and SEM of three independent experiments are shown on the *y*-axis.

To address the question of whether the γH2AX and pATM foci formation associated within the same regions of DNA damage reflect the true DSBs, we analyzed their co-localization. For the acute irradiation, the percentage of co-localized foci increased with the dose from 43% in the control to 67% in the 300 mGy group (Figure 3). In contrast, the fraction of co-localized foci upon chronic irradiation fluctuated near the basal level within the dose range of 30–160 mGy, followed by a non-significant (*p* = 0.068) increase up to 60% at a dose of 300 mGy. This data may indicate a low level of formation of true DSBs during a chronic low dose-rate IR exposure.

### 2.5. Post-irradiation Kinetics of γH2AX and pATM Foci Numbers

**Figure 4:**
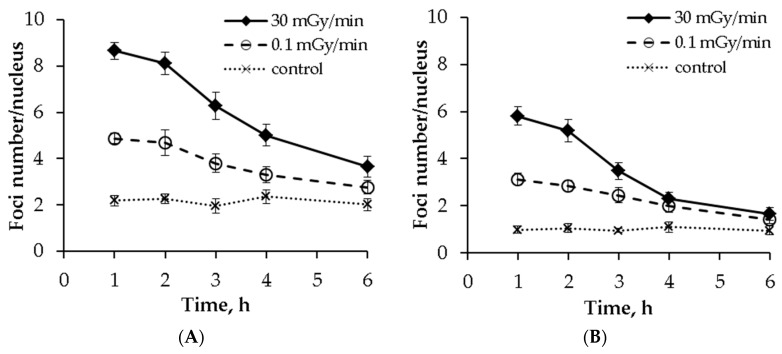
Post-irradiation kinetics of (**A**) γH2AX and (**B**) pATM foci in MSCs exposed either acutely or chronically to γ-radiation. The mean and SEM of three independent experiments are shown.

To evaluate the DSB repair capacity in MSCs upon acute vs. low dose-rate irradiation, we scored γH2AX and pATM at 0, 1, 2, 3, 4 and 6 h after cessation of irradiation delivering a total dose of 300 mGy. The resulting kinetics curves are presented in Figure 4.

It can be seen that the curves for the γH2AX foci were similar for the acute and chronic exposures (Figure 4A). By 6 h, about 70% of the γH2AX foci disappeared, with no statistical difference in the number of residual foci between the two exposure types. The half-life of DSB repair calculated for the γH2AX foci were 2.35 h and 2.44 h for the acute and chronic irradiations, respectively. A larger difference between the two exposure modes was seen when the pATM foci repair was analyzed (Figure 4B). Upon acute exposure, about 25% and 14% of foci still remain at 4 and 6 h, respectively. For the chronic exposure, ~40% and 21% of pATM foci were present at 4 and 6 h, respectively. This indicates a longer half-life of the pATM foci repair for chronic compared to acute irradiation, 2.14 h vs. 1.64 h. However, the difference in the absolute number of residual pATM foci was not significant between the two irradiation modes.

## 3. Discussion

The results of our study showing substantial differences in DNA damage dose-responses to chronic vs. acute irradiation are relevant to the ongoing controversy that surrounds the use of the LNT model in practical radiation protection applications. Radiobiological knowledge that is behind the current radiation protection system has been generated using predominantly acute radiation exposures, including key pioneering experiments that laid the foundation for the LNT model [30]. For a long time, the model has not accounted for the well-known sparing effect (e.g., routinely accounted for in cancer radiotherapy by fractionating the total therapeutic dose) seen for chronic exposures, which are arguably the most common type of human exposure to low-dose radiation. Eventually, the lower biological efficiency of low doses and dose-rates has found its way into the radiation protection system in the form of a DDREF that was arbitrarily given the value of 2, meaning essentially that low doses (<100 mGy) and low dose-rates (<0.1 mGy/min) are 2-fold less effective in causing relevant health detriments, i.e., cancer [2]. However, the DDREF of 2 has since been criticized because radiobiological and epidemiological studies have produced a range of DDREF estimates from 2 to 20 [31]. Interestingly, the World Health Organization’s report on the Fukushima accident used a DDREF of one, which may have led to overestimating cancer risks in exposed populations [32].

Our results showing flatter dose-response curves for the γH2AX and pATM foci induction in MSCs exposed to low dose-rate irradiation when compared to cells exposed acutely (Figure 1 and Figure 2) suggest a lower efficiency in producing DSB by chronic exposure. To quantify the difference in DSB formation by low dose-rate vs. acute irradiation, we calculated the number of foci per unit dose and showed that for the γH2AX and pATM foci the chronic exposure was less effective 2.0–5.5-times and 2.2–10.4-times, respectively (Table 2 and Table 3). In general, these calculations support the use of a DDREF of >2 in estimating the health risks upon exposure to low dose-rate radiation. It is also worth pointing out that the conversion of DNA damage into mutations and further into cancer is thought to be non-linear due to a great variety of defense mechanisms ranging from cell-intrinsic factors, such as inducible DNA repair, to cell-extrinsic factors, such as immune surveillance [33,34]. Addressing the controversy over the LNT vs. threshold model, we assessed the data for the γH2AX and pATM foci formation upon low dose-rate exposures for the goodness of fit using either a linear or a “hockey stick” model (Figure 1B and Figure 2B). When the first three data points representing doses from 0 to 160 mGy were used, a linear fit with a nil slope (flat line or “plateau) could not be rejected for both the γH2AX (*p* = 0.28) and pATM foci (*p* = 0.51). Fitting the entire sets of data showed that both linear and threshold models cannot be rejected. This was true for both the γH2AX and pATM foci data sets. However, it may be pointed out that a “hockey stick” model resulted in a slightly better fit. Consistent with the current results, an inefficient DNA repair foci formation was demonstrated in our previous studies using Chinese hamster V79 cells at a dose-rate of 1 mGy/min [6]. It is tempting to speculate that the indications of a threshold or a “plateau” for dose-response curves for the γH2AX and pATM foci formation upon chronic radiation exposure reflect the real biological processes and can be statistically validated if more data points are included in future studies.

In contrast to chronic irradiation, MSCs exposed to acute irradiation demonstrated linear dose-responses for the γH2AX and pATM foci formation (Figure 1 and Figure 2). The lowest dose of 30 mGy produced significant increases, consistent with a commonly accepted notion that only a few tens of mGy are required to elicit a DSB repair response [35]. The co-localization of γH2AX and pATM foci is generally thought to reflect “true” or physical DSBs that are initially produced by IR [36,37]. The dose-response reconstructed for the co-localized γH2AX and pATM foci (Figure 3) upon acute irradiation followed a linear increase with the dose, unlike the curve observed for MSCs exposed to a low dose-rate irradiation. The latter did not produce statistically significant increases in the number of “true” DSBs. The poor co-localization of γH2AX and pATM foci upon chronic irradiation may suggest the phosphorylation of H2AX carried out by other kinases like ATR or DNA-PKs [38]. It is worth nothing that such phosphorylation is known to occur by ATR in the presence of replication errors and that it involves HR factors, such as Rad51. Consistent with this, the preferential accumulation of Rad51 foci was observed in cells exposed to protracted irradiation and may suggest a more accurate DSB repair via HR [7,10].

However, the simplest mechanistic explanation for the shape of the dose-response curves following the chronic irradiation is the hypothesis of the equilibrium between the formation of DSBs and their repair occurring simultaneously during the protracted exposure time. This implies that DSB repair sensors are capable of detecting the very low rate of radiation-induced DSBs. Such a capability was suggested to play a key role in defining long-term outcomes following low dose-rate radiation exposures [39], and evidence exists that the ATM-dependent DSB signaling machinery may not be capable of detecting damage upon low dose-rate exposures causing an increased cell killing per dose unit [40]. Our results on the kinetics of DSB foci comparing acute vs. chronic exposures demonstrate that DSB repair indeed occurs following the low dose-rate exposure (Figure 4). This is consistent with prevailing evidence showing that DSB repair can indeed act on DSB produced by low dose-rate irradiation. Thus, an efficient DSB repair was demonstrated for human fibroblasts exposed to γ-irradiation at 0.3 mGy/min in vitro [41] and in mouse studies in vivo [42,43,44]. Such an inducible DSB repair was proposed to reduce the chance of spontaneous lymphomas and increase the life expectancy of mice [45]. The rate of endogenous production of single-strand DNA lesions (SSLs) was estimated to equal one caused by irradiation at a dose rate of ~5 mGy/min, which is 50-fold higher than that used in the present study [46]. Such SSLs can lead to stalled replication forks followed by the formation of DSBs [47]. Presumably, these DSBs are repaired by a more accurate mechanism of HR and do not endanger the cell fate [48]. Last, evidence obtained in human in vivo or ex vivo low dose radiation exposures supports the notion of both non-linear dose-responses for DSB formation and the inducibility of DSB repair mechanisms [49,50,51].

In summary, our results show that human MSCs exposed to low dose-rate γ-irradiation produce significantly lower numbers of DSB repair foci compared to acute γ-irradiation. The dose-response curves for DSB repair foci are a better fit with a non-linear “hockey stick” model and contain either a plateau (γH2AX and pATM foci) or a threshold (for “true” DSBs). The efficiency of foci induction by chronic irradiation was 2–10 fold lower than that of acute irradiation, providing support for the DDREF value that is greater than the one currently used. It is equally important that these results provide a scientific basis for the improvement of radiation protection limits to be used in radiation therapy (e.g., protection of healthy tissues), diagnostic imaging, as well as environmental exposures after radiation accidents. Considering the substantial role that human stem cells have recently been shown to play in carcinogenesis and the sparsity of radiobiological data obtained in such cells exposed to low doses, our study generated important novel knowledge. These findings help to understand the biology of human MSCs’ responses to low dose-rate radiation exposures and relate this to long-term health related outcomes. These outcomes, as evidenced in our results, are unlikely to follow a simple linear relationship on dose as predicted by the currently used LNT model. Future research examining these outcomes, such as differentiation capacity, mutational burden and tumorigenicity of MSCs exposed to low dose-rate exposures, is therefore warranted and may be built upon the results presented here.

## 4. Materials and Methods

### 4.1. Cell Culture

Primary human bone marrow MSCs (passage 5–6) were purchased from «Biolot» (Russia). For the immunophenotypic characterization, cells were stained with the panels of antibodies against the following surface markers: CD3, CD13, CD14, CD19, CD25, CD29, CD31, CD34, CD38, CD44, CD45, CD69, CD73, CD90, CD105, CD106, CD166 and HLA-DR (Becton Dickinson, Franklin Lakes, USA). The expression of the surface markers was then analyzed using a BD FACS FACSCalibur (Becton Dickinson Bioscience, USA) flow cytometer. The resulting expression profiles revealed high expression levels (>60% positive cells) for CD90, CD105, CD166, CD44, CD73, medium levels (30–60%) for CD13, CD29 and CD69, and very low levels (<5%) for CD45, CD34, CD133, CD3, CD19, CD25, CD38, CD45, CD106, and CD31 markers. This immunophenotype was consistent with the reported immunophenotype for MSCs [52] and did not change in the course of the experiment. The cells were cultured for two passages in low-glucose Dulbecco’s Modified Eagle Medium (DMEM) (1 g/L glucose) (Thermo Fisher Scientific, Waltham, MA, USA) containing 10% fetal bovine serum (Thermo Fisher Scientific, USA) under the standard conditions in a CO_2_ incubator (37 °C, 5% CO_2_). The culture medium was changed every three days.

### 4.2. The Irradiation Sources and Dosimetry

Chronic irradiation was carried out using the gamma facility “Panorama” (^137^Cs γ-radiation with energy 661.66 keV) that has previously been described for its use in long-term irradiation experiments [53]. In our study, the cumulative absorbed doses were 30, 100, 160, 240 and 300 mGy. The dose rate was 0.1 mGy/min. The radiation field produced in the gamma facility was characterized using the universal dosimeter DKS-101 (Politekhform-M, Moscow, Russia). The dosimeter is capable of measuring absorbed doses in the range of 4 × 10^−2^–4 × 10^4^ mGy and dose rates in the range of 0.25–1.2 × 10^6^ mGy/min. The accuracy of the measurements was less than 6% when the dose rate was >3 mGy/min and the absorbed dose was >6 mGy. The kit included the farmer type camera BMK-06 (SPC Doza, Moscow, Russia) with a sensitive volume of 0.6 cm^3^ (the energy of the detected photon radiation was 0.05–50 MeV). The measurement of the absorbed dose was carried out directly in a CO_2_-incubator. Acute irradiations were carried out in the therapeutic facility «Louch» using gamma rays of a ^60^Co source with an average energy of 1.25 MeV. The dose-rate was 30 mGy/min, and the absorbed doses matched the cumulative doses of the chromic irradiation: 30, 100, 160, 240 and 300 mGy. Gamma-radiation dosimetry was performed using DKS-101 (SPC Doza, Moscow, Russia) and Unidos (PTW-Freiburg, Freiburg, Germany) with an ionizing chamber type 30010 (sensitive volume of 0.6 cm^3^). The total error of the gamma radiation measurements was 5–6% and included the error of the nuclear constants, geometric quotients and detector efficacy.

### 4.3. Cell Irradiation

The MSCs were grown on glass coverslips placed inside 35-mm Petri dishes (Corning Inc., New York, NY, USA) at a density of about 2 × 10^3^ cells per dish. The long-term irradiation of the cell cultures in a CO_2_-incubator at a temperature of 37 °C in the presence of 5% CO_2_ was carried out in the “Panorama” Gamma-ray facility. The duration of exposure was from 5 to 50 h. The duration of acute exposures of cells in 35-mm Petri dishes was from 1 to 10 min. The experiments were repeated three times with four technical replicates for each radiation dose.

### 4.4. Foci Detection and Analysis

The cells were fixed on coverslips in 4% paraformaldehyde in PBS (pH 7.4) for 20 min at room temperature, followed by two rinses in PBS and permeabilization with 0.3% Triton-X100 (in PBS, pH 7.4), supplemented by 2% bovine serum albumin (BSA) to block non-specific antibody binding. The fixed-permeabilized cells were then incubated for 1 h at room temperature with a primary rabbit monoclonal antibody against γH2AX (dilution 1:200, clone EP854(2)Y, Merck-Millipore, Burlington, VT, USA) and a primary mouse monoclonal antibody against the phosphorylated ATM protein (dilution 1:200, clone 10H11.E12, Merck-Millipore, Burlington, VT, USA) diluted in PBS with 1% BSA. After several rinses with PBS, the cells were incubated for 1 h with secondary goat anti-mouse IgG (H+L) antibodies (Alexa Fluor 488-conjugated. dilution 1:600; Merck-Millipore, Burlington, VT, USA) and goat anti-rabbit (rhodamine-conjugated. dilution 1:400; Merck-Millipore, USA) diluted in PBS (pH 7.4) with 1% BSA. Then, the coverslips were rinsed several times with PBS and mounted on microscope slides with ProLong Gold medium (Life Technologies, Waltham, MA, USA) supplemented with DAPI for DNA counter-staining. The slides were examined and imaged using a Nikon Eclipse Ni-U microscope (Nikon, Tokyo, Japan) equipped with a high definition camera ProgRes MFcool (Jenoptik AG, Jena, Germany). The filter sets used were UV-2E/C (340–380 nm excitation and 435–485 nm emission), B-2E/C (465–495 nm excitation and 515–555 nm emission) and Y-2E/C (540–580 nm excitation and 600–660 nm emission). The foci were enumerated manually, with 300–400 cells scored per data point.

### 4.5. Statistical Analysis

The Statistica 8.0 software (StatSoft) was used for the statistical and mathematical analyses of the data. The results represent the means of three independent experiments ± standard error of the mean. The statistical significance was tested using the Student *t*-test.

Linear and hockey stick [54] hypotheses were tested as follows: 10,000 artificial datasets were generated from the normal distribution with the mean gained from the current model and standard error from the experimental data. For both the stochastic and experimental datasets, the total sum of square errors was estimated. The *p*-value is defined as the probability that the total sum of squares for the random dataset would be greater than that for the observed one.

## Figures and Tables

**Table 1:** Relative increase * in γH2AX foci number (I_REL_) depending on dose rate.

Dose Rate, mGy/min	Dose, mGy				
	30	100	160	240	300
0.1	1.28 ± 0.11	1.43 ± 0.18	1.32 ± 0.20	1.93 ± 0.36	2.23 ± 0.30
30.0	1.55 ± 0.27	2.07 ± 0.33	2.78 ± 0.45	3.27 ± 0.60	4.05 ± 0.50

* I_REL_ = I_Di_/I_0_, where I_REL_ is a relative increase in the number of γH2AX foci for a certain dose, I_Di_ and I_0_—values of the number of γH2AX foci for the dose “i”, and for the control group, respectively.

**Table 2:** Normalized coefficient (K) * of the absolute yield of DSB per unit of radiation dose (%).

Dose Rate, mGy/min	Dose, mGy				
	30	100	160	240	300
0.1	2.03 ± 0.74	0.95 ± 0.37	0.44 ± 0.26	0.85 ± 0.30	0.90 ± 0.19
30.0	4.01 ± 1.85	2.34 ± 0.65	2.44 ± 0.54	2.07 ± 0.48	2.23 ± 0.30

* K = (I_Di_ − I_0_)/Di·100 (%), where K is the absolute yield of DSB per unit of radiation dose, Di—irradiation dose, I_Di_ and I_0_—values of the γH2AX foci number following dose “i” and in control, respectively.

**Table 3:** Normalized coefficient (K) * of the absolute yield of DSB per unit of radiation dose (%).

Dose Rate, mGy/min	Dose, mGy				
	30	100	160	240	300
0.1	0.98 ± 1.22	0.59 ± 0.34	0.16 ± 0.27	0.36 ± 0.16	0.67 ± 0.16
30.0	2.18 ± 1.35	1.60 ± 0.37	1.62 ± 0.35	1.56 ± 0.18	1.66 ± 0.26

* K = (I_Di_ − I_0_)/Di·100 (%), where K is the absolute yield of DSB per unit of radiation dose, Di—irradiation dose, I_Di_ and I_0_—values of the pATM foci number following dose “i” and in control, respectively.

## References

[1] Ruhm W., Eidemuller M., Kaiser J.C. (2017). Biologically-based mechanistic models of radiation-related carcinogenesis applied to epidemiological data. Int. J. Radiat. Biol..

[2] Valentin J., International Commission on Radiological Protection (2007). The 2007 Recommendations of the International Commission on Radiological Protection.

[3] Mladenov E., Magin S., Soni A., Iliakis G. (2016). DNA double-strand-break repair in higher eukaryotes and its role in genomic instability and cancer: Cell cycle and proliferation-dependent regulation. Semin. Cancer Biol..

[4] Hoeijmakers J.H. (2001). Genome maintenance mechanisms for preventing cancer. Nature.

[5] van Gent D.C., Hoeijmakers J.H., Kanaar R. (2001). Chromosomal stability and the DNA double-stranded break connection. Nat. Rev. Genet..

[6] Kotenko K.V., Bushmanov A.Y., Ozerov I.V., Guryev D.V., Anchishkina N.A., Smetanina N.M., Arkhangelskaya E.Y., Vorobyeva N.Y., Osipov A.N. (2013). Changes in the number of double-strand DNA breaks in chinese hamster v79 cells exposed to gamma-radiation with different dose rates. Int. J. Mol. Sci..

[7] Osipov A.N., Grekhova A., Pustovalova M., Ozerov I.V., Eremin P., Vorobyeva N., Lazareva N., Pulin A., Zhavoronkov A., Roumiantsev S. (2015). Activation of homologous recombination DNA repair in human skin fibroblasts continuously exposed to x-ray radiation. Oncotarget.

[8] Shibata A. (2017). Regulation of repair pathway choice at two-ended DNA double-strand breaks. Mutat. Res..

[9] Brooks A.L., Hoel D.G., Preston R.J. (2016). The role of dose rate in radiation cancer risk: Evaluating the effect of dose rate at the molecular, cellular and tissue levels using key events in critical pathways following exposure to low let radiation. Int. J. Radiat. Biol..

[10] Tsvetkova A., Ozerov I.V., Pustovalova M., Grekhova A., Eremin P., Vorobyeva N., Eremin I., Pulin A., Zorin V., Kopnin P. (2017). Gammah2ax, 53bp1 and rad51 protein foci changes in mesenchymal stem cells during prolonged x-ray irradiation. Oncotarget.

[11] Pustovalova M., Astrelina capital Te C., Grekhova A., Vorobyeva N., Tsvetkova A., Blokhina T., Nikitina V., Suchkova Y., Usupzhanova D., Brunchukov V. (2017). Residual gammah2ax foci induced by low dose x-ray radiation in bone marrow mesenchymal stem cells do not cause accelerated senescence in the progeny of irradiated cells. Aging.

[12] Li P., Gong Z., Shultz L.D., Ren G. (2019). Mesenchymal stem cells: From regeneration to cancer. Pharmacol. Ther..

[13] Papaccio F., Paino F., Regad T., Papaccio G., Desiderio V., Tirino V. (2017). Concise review: Cancer cells, cancer stem cells, and mesenchymal stem cells: Influence in cancer development. Stem Cells Transl. Med..

[14] Zheng Y., Wang G., Chen R., Hua Y., Cai Z. (2018). Mesenchymal stem cells in the osteosarcoma microenvironment: Their biological properties, influence on tumor growth, and therapeutic implications. Stem Cell Res. Ther..

[15] La Noce M., Paino F., Mele L., Papaccio G., Regad T., Lombardi A., Papaccio F., Desiderio V., Tirino V. (2018). Hdac2 depletion promotes osteosarcoma’s stemness both in vitro and in vivo: A study on a putative new target for cscs directed therapy. J. Exp. Clin. Cancer Res. CR.

[16] Yao D., Dai C., Peng S. (2011). Mechanism of the mesenchymal-epithelial transition and its relationship with metastatic tumor formation. Mol. Cancer Res. MCR.

[17] Xiang C., Chen J., Fu P. (2017). Hgf/met signaling in cancer invasion: The impact on cytoskeleton remodeling. Cancers.

[18] Della Corte C.M., Fasano M., Papaccio F., Ciardiello F., Morgillo F. (2014). Role of hgf-met signaling in primary and acquired resistance to targeted therapies in cancer. Biomedicines.

[19] Andrzejewska A., Lukomska B., Janowski M. Mesenchymal Stem Cells: From Roots to Boost. Stem Cells.

[20] Gomes J.P.A., Assoni A.F., Pelatti M., Coatti G., Okamoto O.K., Zatz M. (2017). Deepening a simple question: Can mscs be used to treat cancer?. Anticancer Res..

[21] Li J.H., Fan W.S., Wang M.M., Wang Y.H., Ren Z.G. (2018). Effects of mesenchymal stem cells on solid tumor metastasis in experimental cancer models: A systematic review and meta-analysis. J. Transl. Med..

[22] Manda K., Kavanagh J.N., Buttler D., Prise K.M., Hildebrandt G. (2014). Low dose effects of ionizing radiation on normal tissue stem cells. Mutat. Res. Rev. Mutat. Res..

[23] Rogakou E.P., Boon C., Redon C., Bonner W.M. (1999). Megabase chromatin domains involved in DNA double-strand breaks in vivo. J. Cell Biol..

[24] Stiff T., O’Driscoll M., Rief N., Iwabuchi K., Lobrich M., Jeggo P.A. (2004). Atm and DNA-pk function redundantly to phosphorylate h2ax after exposure to ionizing radiation. Cancer Res..

[25] Burma S., Chen B.P., Murphy M., Kurimasa A., Chen D.J. (2001). Atm phosphorylates histone h2ax in response to DNA double-strand breaks. J. Biol. Chem..

[26] Bakkenist C.J., Kastan M.B. (2003). DNA damage activates atm through intermolecular autophosphorylation and dimer dissociation. Nature.

[27] Flegal M., Blimkie M.S., Wyatt H., Bugden M., Surette J., Klokov D. (2015). Measuring DNA damage and repair in mouse splenocytes after chronic in vivo exposure to very low doses of beta- and gamma-radiation. J. Vis. Exp..

[28] Blimkie M.S.J., Fung L.C.W., Petoukhov E.S., Girard C., Klokov D. (2014). Repair of DNA double-strand breaks is not modulated by low-dose gamma radiation in c57bl/6j mice. Radiat. Res..

[29] Osipov A.N., Pustovalova M., Grekhova A., Eremin P., Vorobyova N., Pulin A., Zhavoronkov A., Roumiantsev S., Klokov D.Y., Eremin I. (2015). Low doses of x-rays induce prolonged and atm-independent persistence of gammah2ax foci in human gingival mesenchymal stem cells. Oncotarget.

[30] Calabrese E.J. (2017). The threshold vs lnt showdown: Dose rate findings exposed flaws in the lnt model part 1. The russell-muller debate. Environ. Res..

[31] Ruhm W., Woloschak G.E., Shore R.E., Azizova T.V., Grosche B., Niwa O., Akiba S., Ono T., Suzuki K., Iwasaki T. (2015). Dose and dose-rate effects of ionizing radiation: A discussion in the light of radiological protection. Radiat. Environ. Biophys..

[32] World Health Organization (2013). Global report on fukushima nuclear accident details health risks. Relev. Epidemiol. Hebd..

[33] Aurengo A., Averbeck D., Bonnin A., LeGuen B., Masse R., Monier R., Tubiana M., Valleron A.J., de Vathaire F. (2005). Dose-Effect Relationships and Estimation of the Carcinogenic Effects of Low Doses of Ionizing Radiation.

[34] Siegel J.A., Sacks B., Pennington C.W., Welsh J.S. (2017). Dose optimization to minimize radiation risk for children undergoing ct and nuclear medicine imaging is misguided and detrimental. J. Nucl. Med. Off. Publ. Soc. Nucl. Med..

[35] Grudzenski S., Raths A., Conrad S., Rube C.E., Lobrich M. (2010). Inducible response required for repair of low-dose radiation damage in human fibroblasts. Proc. Natl. Acad. Sci. USA.

[36] Horn S., Barnard S., Rothkamm K. (2011). Gamma-h2ax-based dose estimation for whole and partial body radiation exposure. PLoS ONE.

[37] de Feraudy S., Revet I., Bezrookove V., Feeney L., Cleaver J.E. (2010). A minority of foci or pan-nuclear apoptotic staining of gammah2ax in the s phase after uv damage contain DNA double-strand breaks. Proc. Natl. Acad. Sci. USA.

[38] Blackford A.N., Jackson S.P. (2017). Atm, atr, and DNA-pk: The trinity at the heart of the DNA damage response. Mol. Cell.

[39] Morgan W.F., Bair W.J. (2013). Issues in low dose radiation biology: The controversy continues. A perspective. Radiat. Res..

[40] Collis S.J., Schwaninger J.M., Ntambi A.J., Keller T.W., Nelson W.G., Dillehay L.E., Deweese T.L. (2004). Evasion of early cellular response mechanisms following low level radiation-induced DNA damage. J. Biol. Chem..

[41] Ishizaki K., Hayashi Y., Nakamura H., Yasui Y., Komatsu K., Tachibana A. (2004). No induction of p53 phosphorylation and few focus formation of phosphorylated h2ax suggest efficient repair of DNA damage during chronic low-dose-rate irradiation in human cells. J. Radiat. Res..

[42] Osipov A.N., Klokov D.Y., Elakov A.L., Rozanova O.M., Zaichkina S.I., Aptikaeva G.F., Akhmadieva A. (2004). Comparison in vivo study of genotoxic action of high- versus very low dose-rate gamma-irradiation. Nonlinearity Biol. Toxicol. Med..

[43] Osipov A.N., Buleeva G., Arkhangelskaya E., Klokov D. (2013). In vivo gamma-irradiation low dose threshold for suppression of DNA double strand breaks below the spontaneous level in mouse blood and spleen cells. Mutat. Res..

[44] Bong J.J., Kang Y.M., Shin S.C., Choi S.J., Lee K.M., Kim H.S. (2013). Differential expression of thymic DNA repair genes in low-dose-rate irradiated akr/j mice. J. Vet. Sci..

[45] Shin S.C., Kang Y.M., Kim H.S. (2010). Life span and thymic lymphoma incidence in high- and low-dose-rate irradiated akr/j mice and commonly expressed genes. Radiat. Res..

[46] Vilenchik M.M., Knudson A.G. (2003). Endogenous DNA double-strand breaks: Production, fidelity of repair, and induction of cancer. Proc. Natl. Acad. Sci. USA.

[47] Zeman M.K., Cimprich K.A. (2014). Causes and consequences of replication stress. Nat. Cell Biol..

[48] Saleh-Gohari N., Bryant H.E., Schultz N., Parker K.M., Cassel T.N., Helleday T. (2005). Spontaneous homologous recombination is induced by collapsed replication forks that are caused by endogenous DNA single-strand breaks. Mol. Cell. Biol..

[49] Beels L., Bacher K., De Wolf D., Werbrouck J., Thierens H. (2009). Gamma-h2ax foci as a biomarker for patient x-ray exposure in pediatric cardiac catheterization: Are we underestimating radiation risks?. Circulation.

[50] Beels L., Werbrouck J., Thierens H. (2010). Dose response and repair kinetics of gamma-h2ax foci induced by in vitro irradiation of whole blood and t-lymphocytes with x- and gamma-radiation. Int. J. Radiat. Biol..

[51] Neumaier T., Swenson J., Pham C., Polyzos A., Lo A.T., Yang P., Dyball J., Asaithamby A., Chen D.J., Bissell M.J. (2012). Evidence for formation of DNA repair centers and dose-response nonlinearity in human cells. Proc. Natl. Acad. Sci. USA.

[52] Dominici M., Le Blanc K., Mueller I., Slaper-Cortenbach I., Marini F., Krause D., Deans R., Keating A., Prockop D., Horwitz E. (2006). Minimal criteria for defining multipotent mesenchymal stromal cells. The international society for cellular therapy position statement. Cytotherapy.

[53] Lychagin A.A., Ulyanenko S.E., Koryakin S.N., Ulyanenko L.N., Chernukha A.E., Pugachev R.M., Brovin A.I. (2018). Methodological support of radiobiological experiments on the “panorama” facility. Radiobiol. Radioecol..

[54] Lutz W.K., Lutz R.W. (2009). Statistical model to estimate a threshold dose and its confidence limits for the analysis of sublinear dose-response relationships, exemplified for mutagenicity data. Mutat. Res..

